# High-resolution cryo-EM reconstructions in the presence of substantial aberrations

**DOI:** 10.1107/S2052252520002444

**Published:** 2020-03-26

**Authors:** Raquel Bromberg, Yirui Guo, Dominika Borek, Zbyszek Otwinowski

**Affiliations:** a Department of Biophysics, The University of Texas Southwestern Medical Center, Dallas, TX 75390, USA

**Keywords:** cryo-EM, axial aberrations, coma, trefoil, resolution, validation, 3D reconstruction and image processing, automation, single-particle cryo-EM, imaging, structure determination

## Abstract

Here, an analysis is performed of how uncorrected antisymmetric aberrations, such as coma and trefoil, affect cryo-EM single-particle reconstruction (SPR) results, and an analytical formula quantifying information loss owing to their presence is inferred that explains why Fourier-shell coefficient-based statistics may report significantly overestimated resolution if these aberrations are not fully corrected. The analysis is validated with reference-based aberration refinement for two cryo-EM SPR data sets acquired with a 200 kV microscope in the presence of coma exceeding 40 µm, and 2.3 and 2.7 Å reconstructions for 144 and 173 kDa particles, respectively, were obtained. The results provide a description of an efficient approach for assessing information loss in cryo-EM SPR data acquired in the presence of higher order aberrations, and address inconsistent guidelines regarding the level of aberrations that is acceptable in cryo-EM SPR experiments.

## Abstract 2

The beam-image shift method accelerates data acquisition in cryo-EM single-particle reconstruction by rapid repositioning of the imaging area, but at the cost of more complex optical aberrations when the shifts are large, even when the coma is compensated for by beam tilt.

## 1. Introduction   

In a typical cryo-EM single-particle reconstruction (SPR) experiment, some aberrations such as defocus are introduced intentionally, while others such as spherical aberration are unavoidable for a given setup (Cheng *et al.*, 2015 ▸; Wade, 1992 ▸; Scherzer, 1936 ▸). The significance of the remaining aberrations is evaluated on a case-by-case basis (Glaeser *et al.*, 2011 ▸; Uhlemann & Haider, 1998 ▸) or, more recently, by correcting for small aberrations using reference-based refinement (Zivanov *et al.*, 2020 ▸).

In the analysis of aberrations in cryo-EM SPR, where in one image we record only a small part of the focal plane, we can use an isoplanatic approximation in which aberrations are represented by convolutions, and in Fourier space they depend only on the angle of the scattered electrons. In phase-contrast illumination mode, as used in cryo-EM SPR, axial aberrations can be divided into two distinct categories depending on the symmetry properties of the image phase shift as a function of a scattering vector. If the phase shift is centrosymmetric, then aberrations will result in modulations of the image power spectrum. If the phase shift is antisymmetric then the power spectrum will not be modulated, because aberrations will not affect the amplitude of the image but only its phase. The lowest order of antisymmetric phase shift is a translation, which owing to the lack of an absolute coordinate system can be set to zero. Antisymmetric aberrations of the next, third order are called axial coma and trefoil, and these are important for cryo-EM SPR data quality in practice (Cheng *et al.*, 2018 ▸; Glaeser *et al.*, 2011 ▸; Uhlemann & Haider, 1998 ▸). The alignment procedures used in cryo-EM SPR minimize coma and trefoil indirectly, for instance by analyzing changes in the image power spectrum owing to interactions between beam tilt and spherical aberration (Uhlemann & Haider, 1998 ▸). The success of this approach and similar approaches requires co-alignment of the optical axes for multiple lenses, and if the alignment procedures are not properly executed, coma and trefoil may be present and affect the quality of the SPR results, and yet only manifest in specialized analyses (Uhlemann & Haider, 1998 ▸; Mastronarde, 2005 ▸; Suloway *et al.*, 2005 ▸; Cheng *et al.*, 2018 ▸; Zivanov *et al.*, 2020 ▸). Furthermore, these and additional, higher order antisymmetric aberrations are induced when data are acquired using the beam-image shift method (Mastronarde, 2005 ▸; Suloway *et al.*, 2005 ▸; Cheng *et al.*, 2018 ▸; Wu *et al.*, 2019 ▸), in which coordinated electronic shifts of an illuminating beam and an image are used to navigate away from the optical axis. We restrict our discussion here to axial aberrations, but with the understanding that these aberrations will have different values in different positions of an optical system for data acquired with the beam-image shift method, except for coma, which can be compensated for by the beam tilt (Wu *et al.*, 2019 ▸; Glaeser *et al.*, 2011 ▸).

The acceleration in cryo-EM data acquisition enabled by the beam-image shift method (Mastronarde, 2005 ▸; Suloway *et al.*, 2005 ▸; Cheng *et al.*, 2016 ▸, 2018 ▸) has resulted in discussions of the best experimental strategies for data collection, approaches to compensate for or to correct for optical aberrations and methods to assess modulation and the loss of signal owing to the presence of uncorrected optical aberrations (Glaeser *et al.*, 2011 ▸; Herzik *et al.*, 2017 ▸; Cheng *et al.*, 2018 ▸; Li *et al.*, 2019 ▸; Wu *et al.*, 2019 ▸). Axial coma can be corrected by applying compensating beam tilt during data collection; however, beam tilting does not correct other aberrations (Glaeser *et al.*, 2011 ▸), and thus the extent to which one can apply beam-image shift without compromising data quality remains open. There are, however, strong indications that higher order aberrations generate a significant loss of information (Wu *et al.*, 2019 ▸). Wu and coworkers showed that in the case of 6 × 6 hole data collection with compensating beam tilt, the loss of resolution for the final reconstructions was up to 0.45 Å in comparison to data collection in which the beam was on the optical axis. In their experiments, the coma was compensated and corrected, and thus the loss of resolution in the final reconstruction was probably caused by uncorrected higher order aberrations.

We found that large values of axial aberrations can be precisely estimated and accurately corrected for, leading to large, case-specific improvements in SPR results. We provide formulae for assessing how the levels of uncorrected coma and trefoil affect the resolution of SPR, and discuss their impact on the validation statistics.

## 2. Materials and methods   

### 2.1. Protein expression, purification and grid preparation   

Glucose isomerase (GI), also called xylose isomerase, from *Streptomyces rubiginosus* was purchased from Hampton Research. Protein slurry was dialyzed three times against an excess of distilled H_2_O and concentrated to ∼40 mg ml^−1^ with an Amicon filter (Borek *et al.*, 2018 ▸).

The HemQ protein was a structural genomics target (Midwest Center for Structural Genomics APC35880). We have solved its X-ray crystallographic structure (PDB entry 1t0t) and recently others have determined its function (Celis *et al.*, 2017 ▸). The expression and purification of the GYMC52_3505 plasmid encoding HemQ in pMCSG7 vector with a Tobacco etch virus (TEV)-cleavable N-terminal His_6_ tag (Stols *et al.*, 2002 ▸) followed a previously established protocol (Kim *et al.*, 2011 ▸). After purification and tag cleavage, the protein was extensively dialyzed against 20 m*M* HEPES pH 7.5 and used at a concentration of ∼28 mg ml^−1^ for grid preparation. The plasmid GYMC52-3505 is available from the DNASU Plasmid Repository (https://dnasu.org).

Cryo-EM grids for both proteins were prepared with an FEI Vitrobot Mark IV. In each case, 3 µl protein solution was applied to the grid at 4°C and 100% humidity followed by 6 s blotting with blot force 20 before the grids were plunged into liquid ethane cooled with liquid nitrogen.

### 2.2. **Data acquisition and analysis**   

The cryo-EM data set for GI was collected using a 200 kV Talos Arctica microscope equipped with a K2 Gatan camera, with a physical pixel size of 0.91 Å. A phase plate was not used and the objective aperture was not inserted. A total of 202 movies with an exposure time of 100 s per movie were collected. Each movie contains 200 frames with an exposure time of 0.5 s per frame and an electron dose of 120 e Å^−2^ per movie (Table 1 ▸).

Both the HemQ-57K and HemQ-45K data sets were also collected in the same alignment conditions on a 200 kV Talos Arctica microscope with a K2 Gatan camera run in super-resolution mode, with physical pixel sizes of 0.72 Å for HemQ-57K and 0.91 Å for HemQ-45K. For HemQ-57K, 268 movies were collected with an exposure time of 40 s per movie. Each movie contains 100 frames with an exposure time of 0.4 s per frame and an electron dose of 90 e Å^−2^ per movie. For HemQ-45 K, 257 movies were collected with an exposure time of 40 s per movie. Each movie contains 100 frames with an exposure time of 0.4 s per frame and an electron dose of 90 e Å^−2^ per movie.

Complete data sets for EMPIAR depositions 10204, 10185 and 10186 were processed as examples of data sets collected at 200 kV. EMPIAR 10185 and EMPIAR 10186 were collected consecutively on the same instrument, with EMPIAR 10185 collected with a traditional setup by moving only the stage and EMPIAR 10186 collected with the beam-image shift method. We performed image-specific correction for coma in both data sets, producing a material improvement in resolution (Table 2 ▸). EMPIAR 10263, data set III, served as an example of a data set collected at 300 kV with a large coma value that was partially corrected by alternative methods.

We processed all data sets with *cisTEM* (Grant *et al.*, 2018 ▸). We modified the *cisTEM* pipeline by adding reference-based refinement of aberrations, including coma and trefoil, as in *JSPR* and *RELION* (Li *et al.*, 2019 ▸; Zivanov *et al.*, 2018 ▸). As discussed in Sections 3 and 4, we performed multiple cycles that included aberration refinement, orientation refinement and creation of a new reference (Grant *et al.*, 2018 ▸). In these cycles, the resolution limit of the data used for the orientation refinement was selected based on manual assessment of the signal to noise (SNR) estimate in *cisTEM* exceeding a threshold value, typically around 4. The data-collection and analysis statistics are summarized in Table 1 ▸. The cryo-EM movies, maps and models used in the data analysis of GI and HemQ proteins have been deposited in the PDB, the EMDB and EMPIAR under codes 6vrs, EMD-21371 and EMPIAR-10360 for GI; 6vsa, EMPIAR-10363 and EMD-21373 for HemQ-57K; and 6vsc, EMPIAR-10362 and EMD-21376 for HemQ-45K. We solved all of the structures using *MOLREP* (Vagin & Teplyakov, 2010 ▸) and refined them with *REFMAC* (Murshudov *et al.*, 2011 ▸) used within *CCPEM* (Wood *et al.*, 2015 ▸; Burnley *et al.*, 2017 ▸; Nicholls *et al.*, 2018 ▸) with manual inspection using *Coot* (Emsley & Cowtan, 2004 ▸; Emsley *et al.*, 2010 ▸).

In the process of modifying *cisTEM*, we introduced many changes in the underlying data structures, which resulted in the development of a separate software package. The package does not rely on the particle-stack concept which is used in other cryo-EM packages. We plan to release it in the future, and it will include the implementation of aberration refinement described above. The module is, however, not ready yet for standalone release owing to its reliance on unusual data structures.

## 3. Results   

CTF determination by power spectrum analysis is an inherent part of high-resolution cryo-EM SPR and provides estimates of the magnitude of symmetric aberrations (Wade, 1992 ▸). However, phase shift does not modulate the power spectrum, so the determination of antisymmetric aberrations has to be performed by other methods (Glaeser *et al.*, 2011 ▸; Zivanov *et al.*, 2020 ▸).

In our analysis of antisymmetric aberrations, we utilize an associative property of pure phase shift that also holds when it is combined with image translation (Hopkins, 1984 ▸). Thus, corrections for the presence of antisymmetric aberrations can be split into separate steps and applied in any order, without loss of accuracy in retrieving information. Consequently, for both large and small magnitudes of antisymmetric aberrations, their impact is defined only by the difference between their true and their assumed or refined values. If their estimates are inaccurate, the difference generates a component of the image phase shift that, after averaging over multiple particles, produces the signal modulation that we analyze here.

Antisymmetric aberration is a convolution with signal, so in the frequency domain the convolution is represented as the multiplication of a signal and a Fourier representation of an aberration. The impact of aberrations on 3D cryo-EM SPR has been theoretically analyzed by considering how images are affected (Uhlemann & Haider, 1998 ▸; Zivanov *et al.*, 2018 ▸). However, cryo-EM SPR relies on averaging multiple particles to increase the SNR. Thus, we investigated how the impact of an aberration will propagate to averaged representations of particles.

To this end, we assume a large number of particles which are randomly oriented on a grid with respect to rotation around the beam axis but with potential preferred orientation dependence on the other two Eulerian angles. Averaging all such particles removes the dependence of the average signal on the angle of the aberration in the microscope frame (Cheng *et al.*, 2018 ▸). Therefore, the final consequence of an aberration is the resolution-dependent modulation of signal amplitude (Cheng *et al.*, 2018 ▸). For each unique projection that is conceptually equivalent to a 2D class average, we can define an in-plane rotational angle of particle orientation φ in the microscope and the difference Δφ between φ and the characteristic direction of a particular antisymmetric aberration (Supplementary Fig. S1). Single particles have no force aligning them with this angle, so we can assume that their distribution is uniform with respect to rotation perpendicular to the grid. We then express the maximum phase shift *a* for a particular aberration and resolution, which for coma and trefoil is
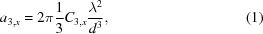
where *d* represents resolution and λ represents electron wavelength. The first index of the aberration coefficients identifies the power dependence on resolution and the other index defines angular periodicity in the microscope frame of the phase shift, and so for coma *x* = 1 and for trefoil *x* = 3 (Barthel, 2007 ▸). Coma can also originate from interactions between beam tilt *b* and spherical aberrations *C*_s_: *C*_3,1_ = 3*b* *C*_s_ so *b* = (*C*_3,1_/3*C*_s_). This representation may be convenient when one is interested in expressing coma with respect to beam tilt rather than coma values.

**Figure 1:**
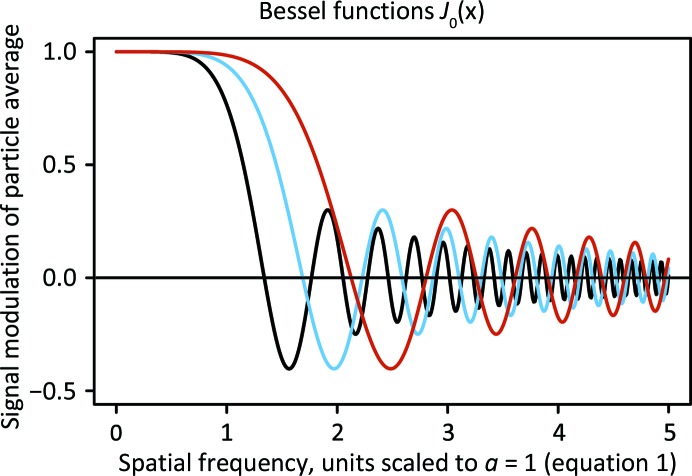
Resolution dependence of third-order aberrations on single-particle reconstruction. The *x* axis represents reciprocal-space resolution, scaled to *a* = 1 for *x* = 1 (equation 1). The *y* axis represents reciprocal-space signal modulation of the reconstruction resulting from averaging the aberration. Three values of aberration coefficients are shown here: black is 1, blue is 0.5 and orange is 0.25.

In weak phase approximation, third-order aberrations can be expressed using terms resulting from the Taylor expansion of a wavefront: exp[−*i*2π(*a*_3,1_cosΔφ_3,1_ + *a*_3,3_cos3Δφ_3,3_)]. To obtain a modulation term for the signal at a given resolution, we average the wavefront over the angular distribution of all possible particles, with the result being the Bessel function of order zero, *J*_0_ (Fig. 1 ▸):
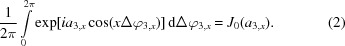
To our knowledge, this analytical result has not been noticed before in assessing aberrations despite having highly important consequences for their analysis. As discussed in detail later, the foremost consequence is that the structure factors of the reconstruction may become anticorrelated from the reality in some resolution shells, an effect which can be missed in the standard, FSC-based half-maps assessment of resolution (Saxton & Baumeister, 1982 ▸; van Heel, 1987 ▸; Scheres & Chen, 2012 ▸), implying much higher resolution than that achieved.

We expect that data acquired with the beam-image shift method will be affected by more than one type of axial aberration. In such a case, aberrations that have the same angular dependence order (second index) but different radial order (first index) will be strongly correlated (Uhlemann & Haider, 1998 ▸) and these correlations have to be considered when values of aberrations are refined. If in refinement the data were to have uniform information content across resolutions, the refinement will be orthogonalized by Zernike polynomials,
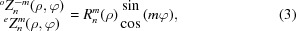
where *R_n_^m^* is the radial function 
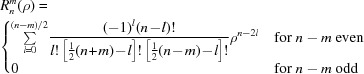
defined for integers *n* and *m* such as that *n* ≥ *m* ≥ 0; φ is the azimuthal angle and ρ is the radial distance, with 0 ≤ ρ ≤ 1. The argument of Zernike polynomial ρ = 1 corresponds to the limiting resolution (Bhatia & Wolf, 1954 ▸).

**Figure 2:**
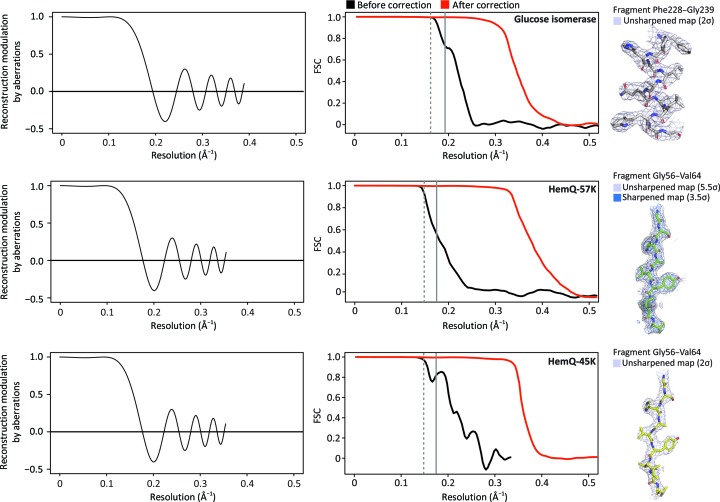
Each row corresponds to one of our three experiments performed with high coma (beam-tilt) values. The left panel shows oscillations of amplitudes caused by uncorrected aberrations calculated with (1) and (2) for each experiment, the middle panel shows FSC plots before (black) and after (red) coma correction and the corresponding final map fragment for three experiments with high coma (beam-tilt) values. The statistics from each experiment are presented in Table 1 ▸. The vertical dotted gray line represents the first zero of the modulation function (2) and the solid line represents the first zero of the modulation function with the assumption that the coma impact was compensated by image translation (5). The resolutions corresponding to the first zero values are listed in Table 2 ▸. The right panel shows fragments of unsharpened maps corresponding to the reconstructed maps.

For coma, the corresponding Zernike polynomial is ρ^3^ − 2/3ρ, with the first term representing coma and the second representing translation. Consequently, at the resolution limit, two-thirds of the coma-produced phase shifts will be compensated for by image translation in refinement. This translation can be executed on the whole image or at the level of particles by shifting their positions in the image by the same value, with such an effect not being noticeable in a typical refinement. However, the compensation factor is reduced from the value of two-thirds because the signal is much stronger at low resolution than at the resolution limit. We determined for the analyzed high-resolution cryo-EM SPR data sets (Table 1 ▸) that translation produced only about a two-fifths compensating contribution, reducing the maximum value of the coma-induced phase shift by a factor of ∼0.6. The compensation owing to resolution dependence explains some of the discrepancies in the literature discussing the acceptable limits of uncorrected coma. For instance, one proposed limit was a π/4 phase shift in the direction of coma distortion, and without translational compensation (Uhlemann & Haider, 1998 ▸; Glaeser *et al.*, 2011 ▸). Using our formula (equation 2), we found that this will preserve *J*_0_(0.6π/4) = ∼0.94 of the original signal, a reduction in the SNR that is barely significant, and so the π/4 limit is too conservative, as was postulated by Cheng *et al.* (2018 ▸). In the analysis of Cheng and coworkers the impact of coma was derived from FSC curves calculated from numerical experiments, in which a particular value of beam tilt (and corresponding coma) was assumed. FSC-based resolution analysis in the presence of uniform large coma can be misleading because the *J*_0_ function oscillates (Fig. 1 ▸). When the signal modulation term defined by *J*_0_ (equation 2) is negative, both halves of the split data are affected, and so the correlation coefficient between the halves is positive even if the resulting reconstruction has a negative correlation with the truth. If the coma causes very strong modulation in the FSC (Li *et al.*, 2019 ▸), then it is easy to recognize the correct resolution limit corresponding to the first zero of the Bessel function *J*_0_ (equations 1 and 2). However, oscillations in the FSC curve may be pronounced or smoothed to different degrees (Fig. 2 ▸), even when data sets with very similar values of coma (Table 2 ▸) were analyzed. The method that we employed from *cisTEM* (Grant *et al.*, 2018 ▸) uses only a smooth spherical mask to calculate FSC curves, and so the oscillations could not have come from a molecular-mask effect (Penczek, 2010 ▸). Thus, in the absence of pronounced oscillations, the resolution limit may be significantly overestimated compared with the consideration based on the first zero of the Bessel function. Consequently, simulation-based procedures relying solely on FSC (Cheng *et al.*, 2018 ▸) may grossly underestimate the significance of coma.

To be more precise, in the case of translation compensation, we introduce the translation using the same notation as for aberration coefficients,
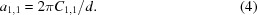
We subtract the phase term with translation to emphasize that this compensates for coma. With such notation, the phase for compensating translation and coma is the same. While the terms *a*_3,1_ and *a*_1,1_ have a different dependence on resolution, the effect is material where the cubic dependence on resolution dominates, so the overall oscillating behavior is similar,
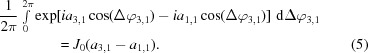
The curves with translation compensation are shown in Fig. 2 ▸.

**Figure 3:**
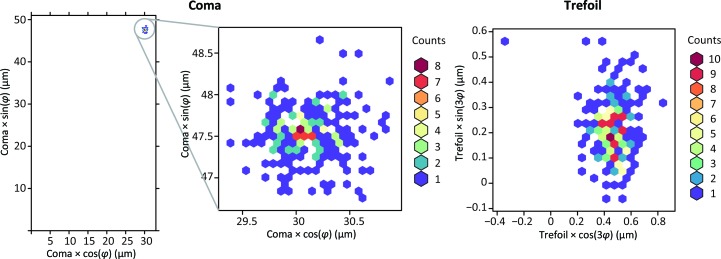
Heat maps for coma and trefoil values refined separately per image from the HemQ-57K data set. The leftmost panel shows tight clustering of the coma values, with the center panel magnifying the region of clustering. The right panel shows the values of trefoil, which for this data set were very low and very consistent across the entire data collection. Dividing coma expressed in micrometres by a factor of 8.1 converts it to the equivalent mrad beam-tilt units assuming *C*_s_ = 2.7 mm.

We tested our approach for two small proteins with particle sizes of 144 and 173 kDa (Fig. 2 ▸, Table 1 ▸), and we obtained reconstructions of 2.32 and 2.70 Å resolution, respectively, in the presence of very high coma, using data acquired with a Talos Arctica 200 kV and K2 Gatan camera. An objective aperture, an energy filter and a phase plate were not used, and the numbers of micrographs and particles in data analysis were moderate (Table 1 ▸). We have not found any detrimental effects for correcting coma, even in cases where the generated phase shift is very large, of the order of 5 × 2π. We reprocessed data from EMPIAR for 200 kV instruments, applying the same aberration estimation approach to data for the larger molecules of the proteasome (700 kDa, EMPIAR 10185 and 10186; Herzik *et al.*, 2017 ▸) and β-galactosidase (430 kDa, EMPIAR 10204; Iudin *et al.*, 2016 ▸) (Table 1 ▸). We refined coma and trefoil independently on each micrograph. We noticed that coma can fluctuate far above refinement uncertainty, and we attribute this observation to differences in the stability of the beam-tilt direction between micrographs. In addition, coma refinement has a strong correlation with overall image shift, affecting the accuracy of coma determination. We found that trefoil on the other hand was remarkably stable and has no significant correlation with other parameters of refinement, so variations in its refined value can be used as a good indicator of statistical uncertainty for third-order aberrations (Fig. 3 ▸).

The problem generated by the presence of large coma has been analyzed in a recent publication (Li *et al.*, 2019 ▸) which described the refinement of EMPIAR 10263 (data set III) with coma refinement performed by *RELION*-3.0 (Zivanov *et al.*, 2018 ▸) and *JSPR* (Guo & Jiang, 2014 ▸; Li *et al.*, 2019 ▸), with the results presented in Fig. 6(*b*) in Li *et al.* (2019 ▸).

We reprocessed this data set and obtained a much tighter clustering of coma values, similar to the clustering observed for our data sets. *RELION*-3.0 (Zivanov *et al.*, 2018 ▸) underestimated coma by a factor of ∼4 compared with our estimate, and the average of the *JSPR* individual refinements per micrograph had a value that was underestimated by 25% compared with our values. However, we used a similar reference-based refinement procedure as *JSPR* (Li *et al.*, 2019 ▸; Guo & Jiang, 2014 ▸) and *RELION*-3.0 (Zivanov *et al.*, 2018 ▸, 2020 ▸), and so the different outcomes are most probably owing to differing refinement schemes (Li *et al.*, 2019 ▸). This was confirmed by a discussion with the author of the publication, who stated that coma refinement was not followed by additional cycles of particle global orientation and coma refinement, unlike in our approach. Thus, the results presented in Fig. 6(*b*) in Li *et al.* (2019 ▸) are consistent with the behavior of our procedure when compared with the results from our first cycle.

We observed that only the presence of a large fraction of incorrect particles or particles with grossly incorrect orientation in a micrograph would bias the coma refinement towards the starting point. When starting with coma refined by the previous cycle, re-refining the particle orientation attenuated the bias in the next cycle, so even the presence of a moderate number of bad particles did not affect the convergence of the procedure (Cash *et al.*, 2020 ▸). We achieved additional improvement, in terms of resolution and the spread of coma values, when we changed the null hypothesis regarding coma from a value of zero to the average of the refined coma values. This more appropriate null hypothesis was applied by taking advantage of the associative properties of coma, which allowed us to apply coma phase-shift correction to the images; therefore, all of the steps in the final round of the data analysis, starting from particle picking, were performed on images corrected by the average value of coma. Subsequent coma refinement, representing a difference from the previous average used in image correction, was still performed independently for all micrographs, but the residual bias resulting from the initial coma value being zero was eradicated. Coma or beam-tilt refinement has to use a reference-based target function, irrespective of implementation. We would expect that small corrections would be equally well characterized by all programs. However, local refinements and restraints may have different convergence ranges depending on the implementation, and this is mostly likely to be the explanation for the differences in results between programs.

Uncorrected aberrations create systematic patterns of phase shift, and if such aberration error is constant across a data set then the reconstruction will also be altered in a systematic way, not only by losing amplitude but also by flipping its sign at some resolutions. In this case, the FSC curve may undergo oscillations, with the first minimum being the effective resolution limit of the result. Oscillations in the FSC curve are recognized as a qualitative sign of problems with the quality of cryo-EM SPR results (Penczek, 2010 ▸). We provide here in (2) an explanation for another possible source of these oscillations. In our glucose isomerase (GI) data acquired with 58 µm coma, corresponding to 7.2 mrad beam tilt, before correcting coma we observed four oscillations in the FSC curve resulting from phase shift (Fig. 2 ▸), with the map interpretability being inconsistent with the FSC-based resolution indicator. Therefore, if FSC oscillations are encountered, refining coma is highly recommended to diagnose the problem, with the potential outcome being substantial reconstruction improvement by correcting the aberration.

## 4. Discussion   

Although instruments may be aligned quite accurately before data collection, our analyses and those of others (Li *et al.*, 2019 ▸; Cheng *et al.*, 2018 ▸; Herzik *et al.*, 2017 ▸; Glaeser *et al.*, 2011 ▸) indicate that coma can vary significantly between images and the extent of the variation is data-set dependent. We do not fully understand why coma varies, but a possible explanation may involve avoiding stage shift for small position changes without beam-tilt adjustment, with the allowed range of such shifts being a user-adjustable parameter. Another possible explanation is small fluctuations in the stability of the objective lens. The reason for this does not necessarily have to be hardware problems, for example temperature fluctuations; it can also arise from the data-collection scheme, as we and others (Wu *et al.*, 2019 ▸) have observed that the beam-image shift approach is not only faster but generates different patterns of experimental instabilities. However, until this problem is further resolved, we recommend refinement of not only overall coma but also coma for individual images. Trefoil typically is not pronounced, but for one pair of EMPIAR data sets (EMPIAR 10185 and EMPIAR 10186), and also in our data sets (not shown), it was highly significant. However, even in these cases only the overall value of trefoil for the data set was important, with an insignificant level of variations between individual images. (2) provides quantification of the impact that uncorrected aberrations have on reconstruction in specific cryo-EM SPR experiments and associated computations. The dependence of SNR on resolution in the presence of uncorrected aberrations described by (2) has characteristic modulations (Fig. 1 ▸) that allow the separation of the impact of aberrations from other effects in reconstruction, consequently guiding experimental strategy.

Correction for antisymmetric aberrations can be performed during reconstruction (Li *et al.*, 2019 ▸; Zivanov *et al.*, 2018 ▸, 2020 ▸), but the consequence of the associative property is that it can just as well be performed at the whole-micrograph level before reconstruction commences. In the presence of large coma, correcting coma at the image level reduces the point-spread function of the imaging system so it may improve particle-masking operations. If the value of these aberrations is known from external calibration then the image-based correction may simplify the application of these corrections to the data without modifying downstream analysis programs.

What affects cryo-EM SPRs is the error in the aberration model used and not the magnitude of the aberrations themselves, at least up to the theoretical limit where the image of a point source (for example due to coma) extends outside the detector. Therefore, it is important to calibrate all aberrations and not only those that affect the power spectrum. This can be accomplished prior to data collection or afterwards by reference-based refinement using the structure being solved. Once appropriate coma calibrations and corrections are used, this means that procedures where the beam is intentionally tilted can be used on a larger scale than those applied until now (Wu *et al.*, 2019 ▸; Cheng *et al.*, 2018 ▸). This can provide limited but additional three-dimensional particle information on top of the projection image without any of the time and precision penalties associated with mechanical rotations. 

## Supplementary Material

PDB reference: glucose isomerase, 6vrs

PDB reference: HemQ-57K, 6vsa

PDB reference: HemQ-45K, 6vsc

EMDB reference: glucose isomerase, EMD-21371

EMDB reference: HemQ-57K, EMD-21373

EMDB reference: HemQ-45K, EMD-21376

Supplementary Figure S1. DOI: 10.1107/S2052252520002444/fq5012sup1.pdf

## Figures and Tables

**Table 1:** Data collection and processing

	GI	HemQ-57K	HemQ-45K	EMPIAR 10185	EMPIAR 10186	EMPIAR 10204
Instrument	Talos Arctica 200 kV					Cryo-Arm 200 kV
Phase plate	No	No	No	No	No	No
Energy filter	No	No	No	No	No	No
Objective aperture	No	No	No	Yes	Yes	Not known
Frames per movie	200	100	100	68	68	49
Electron dose (e Å^−2^ per frame)	0.7	0.9	0.9	0.99†	1.0†	1.38
Exposure time (s per frame)	0.5	0.4	0.4	0.25	0.25	Not known
K2 super-resolution mode	No	Yes	Yes	Yes	Yes	No
Detector pixel size (Å)	0.91	0.72	0.91	0.91	0.91	0.885
Data pixel size (Å)	N/A	0.36	0.455	0.455	0.455	N/A
Movies collected/deposited	202	268	257	315	260	2161
Movies used for processing	149	258	173	315	260	415
Molecular weight (kDa)	173	144	144	659	659	465
Particle symmetry	*D*2	*C*5	*C*5	*D*7	*D*7	*D*2
Total picked particles	114522	156210	236091	109695	91186	157513
Particles after 2D averaging	85527	145966	174776	No 2D classification	No 2D classification	82721
Particles used in refinement	61909	81302	129446	85847	78689	52340

† Count-based estimation.

**Table 2:** The resolution without and with correction for coma for the analyzed data sets

	GI	HemQ-45K	HemQ-57K	EMPIAR 10204	EMPIAR 10185	EMPIAR 10186
Reported resolution (Å)	NA	NA	NA	NA	3.1	3.3
FSC_0.143_-based resolution before correction (Å)	4.1	3.8	4.3	2.5	3.1	3.2
FSC_0.143_-based resolution after correction (Å)	2.7	2.6	2.3	2.5	2.5	2.4
Coma (µm)/beam tilt (mrad)	42.7/5.3	56.9/7.0	56.2/6.9	0.36–5.22/0.13–1.93	0.89–3.57/0.33–1.32†	1.41–4.61/0.52–1.70
Trefoil (µm)	0.62	0.79	0.49	0.01–0.56	0.76–0.98	0.93–1.07
Resolution at the first oscillation of *J*_0_ with 40% compensation	5.2	5.7	5.7	ND	ND	ND

† EMPIAR 10185 and 10186 were collected consecutively and share the same stable value of trefoil. EMPIAR 10185 was collected with stage shift and has similar coma variation as EMPIAR 10204. EMPIAR 10186 was collected with beam-image shift that induced additional coma variation.
