# Supplementary material for: High-resolution cryo-EM reconstructions in the presence of substantial aberrations
Source: IUCrJ. 2020 Mar 26;7(Pt 3):445–52. doi: 10.1107/S2052252520002444 (PMC7201289; doi:10.1107/S2052252520002444)
Supplement: Supplementary file 1 [file m-07-00445-sup1.pdf]

IUCrJ

Volume 7 (2020)

Supporting information for article:

High-resolution cryo-EM reconstructions in the presence of substantial aberrations

Raquel Bromberg, Yirui Guo, Dominika Borek and Zbyszek Otwinowski

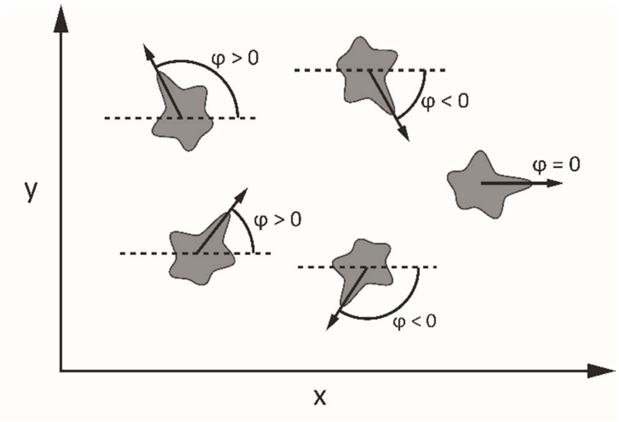

Figure S1 Definition of the orientation angle φ for particles coming from the same projection. This angle is not affected by forces generating preferred orientation in the typical setup of the sample grid being perpendicular to the beam.
